# Modulation of Peripheral Mast Cell and Brain Microglia Axis via Kinase Inhibition

**DOI:** 10.3390/metabo15030194

**Published:** 2025-03-11

**Authors:** Xiaoguang Liu, Michaeline Hebron, Kaluvu Balaraman, Louis Ballard, Kimberly Liu, Max Stevenson, Charbel Moussa

**Affiliations:** 1 Translational Neurotherapeutics Program, Laboratory for Dementia and Parkinsonism, Department of Neurology, Georgetown University Medical Center, Washington, DC 20057, USA; mlh88@georgetown.edu (M.H.); lcb105@georgetown.edu (L.B.); kimbliu06@gmail.com (K.L.); mes407@georgetown.edu (M.S.); 2 Chemistry Department & Medicinal Chemistry Shared Resource, Georgetown University, Washington, DC 20057, USA; bala04chem05@gmail.com

**Keywords:** c-KIT/c-Abl (Abelson) inhibitor, autophagy, pTau, inflammation, neurodegeneration

## Abstract

**Background/Objectives:** Kinase inhibition is a hot therapeutic strategy for several human diseases, including neurodegeneration. Tyrosine kinase c-KIT activates peripheral mast cells, while other kinases including Abelson (c-Abl) promotes autophagy and FYN mediates Tau phosphorylation. We synthesized a novel broad kinase inhibitor (BK40196) and investigated its effects on tau hyper-phosphorylation, cell loss, inflammation and behavior in transgenic rTg4510 and TgAPP (TgSwDI) mice. **Methods:** Drug synthesis and investigation of the pharmacokinetics and pharmacodynamics effects of BK40196 on behavior, protein levels, mast cells and microglial activity in vivo. **Results:** We synthesized a novel kinase inhibitor (BK40196) that exhibited high brain penetration and a potentially wide therapeutic dose. BK40196 is a dual c-KIT/c-Abl (Abelson) inhibitor but also displays binding affinity to other kinases, including fused in sarcoma (SRC) and FYN. BK40196 induces autophagy in vitro and limits the maturation of mast cells in vitro and in vivo. BK40196 significantly reduces the levels of hyper-phosphorylated tau and attenuates cell loss, while improving motor, cognitive and behavioral (anxiety) functions in models of neurodegeneration. BK40196 reduces microglial activity and the levels of brain tryptase in parallel with mast cell activation. **Conclusions:** BK40196 inhibits c-Kit and may play an important role in peripheral and central immunity via mast cells and microglia, respectively, and induces synergistic mechanisms through anti-inflammation and protein clearance that are mutually beneficial to alleviate neurodegenerative pathology. BK40196 is a potential candidate for the treatment of human tauopathies.

## 1. Introduction

Targeting kinases is a key strategy in therapeutic development for human diseases. Tyrosine kinase (TK) is one class of enzyme which has been scientifically validated and clinically exploited as a therapeutic option in cancer and is currently being investigated in neurodegenerative diseases [1,2]. Many TK enzymes are activated or upregulated in postmortem brains from Alzheimer’s disease (AD) and Parkinson’s disease (PD) patients [3,4,5]. TK activity has been implicated in AD pathology, as several downstream substrates may lead to cytokine production and autophagic defects and manifest in the accumulation of misfolded amyloid proteins [4,6,7,8]. Evidence of TKs as desirable, feasible and viable targets in neurodegenerative diseases is mounting and pharmacological inhibition of TKs, such as Abelson (c-Abl), Discoidin Domain Receptor-1 (DDR1), c-Kit and fused in sarcoma (Src), mitigates neurodegeneration in neurodegeneration models [3,4]. Nilotinib, an c-Abl/DDR1 inhibitor, reduces pathological markers such as amyloid and tau in the plasma, brain (via positron emission tomography) and cerebrospinal fluid (CSF) of AD patients [9] and may stabilize long-term motor and cognitive decline in PD patients. Phase 2a shows that bosutinib, a c-Abl/Src inhibitor, significantly increases dopamine and reduces alpha-synuclein levels and improves activities of daily living in patients with Dementia with Lewy Bodies (DLB) [10,11]. Masitinib, a c-KIT inhibitor, is currently in phase 3 clinical trials for AD [12] and multiple sclerosis [13].

Current FDA-approved TKIs for oncology all display poor brain penetrance and considerable toxicity, including myelosuppression and cardiovascular toxicity via QTc prolongation, while evidence suggests that kinase inhibition (via lower drug dosage) provides an effective means of alleviating neurodegenerative pathologies [3,10,11]. We synthesized a small library of new chemical entities (NCEs), including BK40196 that readily enters the CNS [14], and we were able to demonstrate that BK40196 primarily targeted c-KIT, c-Abl and Src family kinases, including FYN. BK40196 can inhibit c-KIT in both the brain and periphery on mast cells, thus modulating mast cell-microglia inflammatory response. BK40196 facilitates autophagy and clearance of amyloid and tau in models of AD and the tauopathies.

## 2. Methods

### 2.1. Synthesis of BK40196

We prepared N-heterocyclic aromatic scaffolds with the goal to improve activity with physicochemical characteristics favorable for brain uptake and crossing of the BBB as we previously reported [14]. N-(3-methoxyphenyl)-2-(m-tolyl)thieno[3,2-b]pyridin-7-amine (BK40195). BK40195 was obtained as a colorless solid in 92% yield (95 mg, 0.276 mmol) from 7-chloro-2-(m-tolyl)thieno[3,2-b]pyridine (78 mg, 0.3 mmol) and m-anisidine (74 mg, 0.6 mmol) in 1 mL of DMSO after 16 h at 100 °C. The cooled reaction mixture was extracted with EtOAc and washed with water. The combined organic layers were dried over sodium sulfate and the solvent was removed in vacuo. The crude product was purified by flash chromatography on silica gel (particle size 40–63 μm) using hexanes–ethyl acetate as mobile phase. Rf = 0.2 (hexanes/EtOAc, 2:1); 1H NMR (400 MHz, Chloroform-d) δ = 8.41 (d, J = 5.6 Hz, 1H), 7.70 (s, 1H), 7.58–7.51 (m, 2H), 7.36–7.29 (m, 2H), 7.21 (d, J = 7.9 Hz, 1H), 6.96 (d, J = 5.6 Hz, 1H), 6.87 (dd, J = 7.9, 2.4 Hz, 1H), 6.83 (dd, J = 7.8, 7.7 Hz, 1H), 6.73 (dd, J = 7.9, 2.5 Hz, 1H), 6.07 (s, 1H), 3.83 (s, 3H), 2.44 (s, 3H); 13C NMR (100 MHz, Chloroform-d) δ = 160.8, 158.6, 148.9, 146.5, 145.6, 140.7, 139.0, 133.7, 130.5, 130.0, 129.1, 127.3, 123.8, 121.7, 120.9, 114.5, 110.1, 108.1, 103.0, 55.5, 21.6; Anal. Calcd. for C21H18N2OS: C, 72.80; H, 5.24; N, 8.09. Found: C, 72.53; H, 5.61; N, 8.19.

3-((2-(*m*-Tolyl)thieno[3,2-*b*]pyridin-7-yl)amino)phenol (BK40196). To a solution of *N*-(3-methoxyphenyl)-2-(*m*-tolyl)thieno[3,2-*b*]pyridin-7-amine (BK40195) (69 mg, 0.2 mmol) in dry dichloromethane (3 mL) was added boron tribromide (4 equiv) at −78 °C under inert atmosphere. The mixture was stirred for 4 h and the reaction temperature was allowed to reach 0 °C. The crude reaction mixture was quenched with 1M HCl, extracted with EtOAc and washed with water. The combined organic layers were dried over sodium sulfate and the solvent was removed in vacuo. The crude product was purified by flash chromatography on silica gel (particle size 40–63 μm) using DCM-MeOH (19:1) as mobile phase. The desired product was obtained as a colorless solid in 97% yield (64 mg, 0.194 mmol). *R_f_* = 0.4 (DCM/MeOH, 9:1); ^1^H NMR (399 MHz, Methanol-*d*_4_) δ = 8.22 (d, *J* = 6.7 Hz, 1H), 7.70 (s, 1H), 7.65 (s, 1H), 7.61 (dd, *J* = 7.5, 2.1 Hz, 1H), 7.40 (dd, *J* = 7.6, 7.6 Hz, 1H), 7.34–7.31 (m, 2H), 6.93 (d, *J* = 6.7 Hz, 1H), 6.88 (m, 1H), 6.85–6.79 (m, 2H), 2.44 (s, 3H); ^13^C NMR (100 MHz, Methanol-*d*_4_) δ = 160.1, 154.7, 153.5, 149.5, 141.1, 140.7, 139.5, 133.3, 132.4, 131.9, 131.7, 130.5, 128.2, 125.0, 117.4, 115.7, 114.9, 113.5, 102.7, 21.3; Anal. Calcd. for C_20_H_16_N_2_OS: C, 72.26; H, 4.85; N, 8.43. Found: C, 72.29; H, 4.97; N, 8.61.

### 2.2. Kinase Screening

We performed the KINOME*scan*™ screening platform (Eurofins DiscoverX Corporation, San Diego, CA, USA) to quantify interactions between BK40196 human kinases and disease relevant mutant variants (450 targets/hits). The kinase active site-directed competition binding assay is independent of ATP and measures thermodynamic interaction or affinities via direct (steric) or indirect (allosteric) binding of BK40196 to the kinase active site and prevents kinase binding to immobilized ligands. Hits are measured via quantitative and ultra-sensitive qPCR as the amount of kinase captured in test (BK40196) versus control samples. BK40196 was screened at 10µM concentration, and screen binding interactions were reported as “% Ctrl” (POC), where lower numbers indicate stronger hits.

E. coli derived from BL21 strain were grown in parallel in 24-well blocks to log-phase and infected (multiplicity of infection = 0.4) with kinase-tagged T7 phage and incubated with shaking at 32 °C until lysis for 90–150 min and the kinases were produced in HEK-293 cells according to manufacturer’s protocol. Test compounds were prepared as 100× stocks in 100% DMSO and all reactions were performed in polypropylene 384-well plates in a final volume of 0.02 mL at room temperature with shaking for 1 h.

### 2.3. In Vitro Mast Cell Analysis

Human mast cells (LUVA cells, Kerafast) were grown in Stempro-34 serum-free media with 10% nutrient supplement, 1% L-glutamine, and 1% pen/strep according to manufacturer’s protocols. Cells were split upon confluence and treated with 100 nM stem cell factor (SCF, Stemcell Technologies) overnight, followed by DMSO or an ascending dose of BK40196 (10 nM–10μM) for additional 48 h. Cells were incubated with two flow-conjugated antibodies (1 μg/mL): PE anti-mouse CD117 (c-KIT) and APC/fire 750 anti-mouse FcεR1α (BioLegend) for 30 min and resuspended in cell staining buffer and analyzed on a Becton Dickinson LSRFortessas flow cytometer.

### 2.4. In Vitro Autophagy Assay

Human SH-SY5Y neuroblastoma cells (Millipore Sigma) were cultured in DMEM with Ham’s F12 (1:1) (ThermoFisher, 11765054) with 10% FBS, 1% PenStrep and 1% L-glutamine. Cells were plated at a density to reach 70–80% confluence at the beginning of every experiment. The cells then were treated with 0.1µM/ 1µM/10µM of BK40196, or DMSO or rapamycin/chloroquine overnight before being harvested and treated with the Autophagy Assay kit (Abcam Cat No ab139484) according to manufacturer’s protocols and analyzed via flow cytometry.

### 2.5. In Vivo Mast Cell Analysis

Animals were euthanized with CO_2_ (3.7 L/minute) in a mouse chamber (6.5″ × 12.5″ × 5.5″) equivalent to approximately 50% flow rate per minute and death was verified by cervical dislocation and the spleen was collected from BK40196-treated and vehicle DMSO-treated TgAPP mice. TgAPP express the neuronally derived human APP gene, 770 isoform, containing the Swedish K670N/M671L, Dutch E693Q and Iowa D694N mutations (TgAPP) under the control of the mouse thymus cell antigen 1, theta, Thy1, and promoter. The spleen was then excised to pieces and mashed over a 40 µm cell strainer and flushed with 10 mL of 1× PBS. Mixtures were then centrifuged at 350× *g* for 5 min at room temperature. Supernatant was removed and pellets were resuspended in 1× PBS at 3 × 10^6^ cells/mL. The cells were then treated with three flow-conjugated antibodies: 647 anti-mouse CD45, PE anti-mouse CD117 (c-KIT) and APC/fire 750 anti-mouse FcεR1α (BioLegend) for 30 min, then treated with 1× RBC lysis buffer (BioLegend #420301) for 20 min and analyzed on a Becton Dickinson LSRFortessas flow cytometer. Percentage of cells positive for c-KIT and FcεR1α were compared.

### 2.6. Animal Euthunasia and Tissue Preparation

Animals were euthanized with CO_2_ (3.7 L/minute) in a mouse chamber (6.5″ × 12.5″ × 5.5″) equivalent to approximately 50% flow rate per minute and death was verified by cervical dislocation for experiments. For Western blot and ELISA sample collection, animals will be cardiac perfused in order to obtain good quality immunohistopathologic assessment under the various treatment conditions according to approved Protocol# 2016-1194. To perform the perfusion, animals are deeply anesthetized with an intraperitoneal injection of sterile solution of ketamine 80–100 mg/kg and xylazine 8 mg/kg. The heavily anesthetized animal is placed on its back on a rack over a sink. A cut is made along the sternum ~3 cm long; that is low to expose the sternum’s end. The end of the sternum is grasped with a 5″ hemostat, and the diaphragm is cut laterally on both sides. The descending aorta is clamped. The hose clamp on the perfusion tube is released so that the fluid is just barely passing out of the cannula tip. The left ventricle of the heart is pierced, and a cannula is inserted and directed up through the left ventricle toward the ascending aorta and clamped to hold the tip of the cannula in place. The right auricle is punctured, allowing the escape of return circulation. Animals are then perfused using a perfusion pump with phosphate-buffered saline (PBS) until the outlet fluid from the right auricle is clear. The brain is removed. For immunohistochemistry, animals were perfused as above, followed by cold 4% paraformaldehyde (PFA) in PBS for at least 20 min and then washed with PBS for 3–4 min. The animal’s skull was removed and the whole brain was dissected out and immersed in fixative (4% PFA in PBS) at 4 °C. For pharmacokinetics, animals were deeply anesthesized with an intraperitoneal injection of a sterile solution of ketamine 100 mg/kg and xylazine 8 mg/kg and blood was rapidly collected by percutaneous cardiac venipuncture and the whole brain was dissected out.

### 2.7. Pharmacokinetic Analysis

The pharmacokinetic (PK) profile of BK40196 was investigated after a single intraperitoneal injection at the dosage of 20 mg/kg or 40 mg/kg BK40196 to male and female 5–6-month-old wild-type C57BL/6 mice. A solution at 5 ng/mL deuterium isotope labeled reference compounds d8-BK40196 was prepared. The solvent used for ISTD dilution was acetonitrile (ACN) and ethyl acetate (EtOAc) (ACN/EtOAc) (200 mL/50 mL). Brain tissue was homogenized in an ice water bath and was prepared using weight accurately on analytical balance with 4 decimals. A volume water (MilliQ) was added by adjustable 100–1000 μL to a concentration at 100 mg brain tissue/mL water. Brain homogenate (25 μL) and plasma sample (25 μL) that was thawed to room temperature and gently mixed were pipetted into an eppendorf tube with lid and 75 μL ISTD added, mixed on vortex, and centrifuged for 5 min at 12,3 RCF. A volume of 25 μL milli-Q water and 75 μL brain or plasma samples were added into a 200 μL PE vial and mixed on vortex and 10 μL were injected via LC-MS/MS.

### 2.8. Determination of Maximal Tolerated Dose and Tissue Toxicity

To determine the maximum tolerated dose of BK40196 and the tissue toxicity, a dose escalation study was performed. Male and female C57B/L6 (6–8-month-old) mice were treated with BK40196 starting from a daily dose of 5 mg/kg intraperitoneally (i.p) for 5 increment days and then we gradually increased the dosage to 10, 20, 30, 40, 50, 60, 70 and up to 80 mg/kg i.p injection.

### 2.9. Sex as a Biological Variable

Sex was not considered a biological variable, as both male and female mice were used and showed the same results.

### 2.10. Transgenic Mice and Treatment

Male and female TgAPP (16–18-month-old) mice were used for the model of AD. Another animal model we used was rTg4510 mice. rTg4510 mice express human P301L Tau and have the Tet-responsive element (TRE or tetO) and mouse prion protein promoter sequences (PrP or Prnp), directing expression of the P301L mutant variant of the human four-repeat microtubule-associated protein tau (4R0NTau P301L). Since rTg4510 may develop age-related neuropathology and behavior impairments, we compared male and female young (5–6-month-old) and old (11–12-month-old) rTg4510 mice.

The mice were treated with an intraperitoneal injection of vehicle (DMSO) or BK40196 at the daily dosage of 20 mg/kg for 3–5 weeks. Mice were age- and sex-matched in their respective treatment cohorts. All mice were sacrificed after treatment.

### 2.11. Rotarod Test

Mice were placed on an accelerating Rotarod (Columbus Instruments, Columbus, OH, USA) equipped with individual timers for each mouse as we previously described.

### 2.12. Elevated Plus Maze Test

This test uses an elevated, plus-shaped (+) apparatus with two open and two enclosed arms. For testing, mice were placed individually in the center of the maze, the number of entries to open arms, the number of entries to closed arms, the time spent in open arms, the time spent in closed arms, total distance traveled, and total number of entries to both open and closed arms during 10 min period were recorded by trained and blinded observers.

### 2.13. Novel Object Recognition

A novel object recognition test was carried out using a 50 × 50 × 50 cm open field arena with clear walls covered by brown construction paper. Mice were habituated to the arena for 5 min two times daily for 3 days. On the fourth day, mice were allowed to familiarize themselves with the two identical objects which were placed in the arena in opposite adjacent corners for 10 min. One object was then replaced with a novel object, and mice were again allowed to freely explore for 10 min, with the approach time toward the novel and familiar objects recorded. The discrimination index was calculated by dividing time spent exploring the novel object by total time exploring both objects and was compared for treated vs. control animals.

### 2.14. Morris Water Maze

The Morris water maze test was performed using a standard circular pool filled with opaque water at around 25 °C. A clear platform was placed at a fixed location just beneath the water surface in one quadrant of the pool. Mice performance was recorded with ANY MAZE software.

### 2.15. Immunohistochemistry

Brains were stored in 4% PFA for 24 h at 4 °C, and then transferred to 30% sucrose at 4 °C for 48 h. 20 µm thick coronal sections were stored at −20 °C. Staining for amyloid-beta plaques was probed with anti-amyloid beta (6E10) (Cat No: 803001, BioLegend, San Diego, CA, USA). c-KIT was stained using an anti-CD117 monoclonal antibody (Cat No: 14-1171-82. Invitrogen, Carlsbad, MA, USA). Iba1 was stained using a rabbit anti-Iba1 antibody (Cat No: 019-19741, Fuji, Japan). Tryptase was stained using a mast cell tryptase recombinant rabbit monoclonal antibody (Cat No: MA5-38007, Invitrogen, MA). Nuclear staining with 4′,6-diamidino-2-phenylindole (DAPI) was performed according to the manufacturer’s protocol (Life Technologies, Miami, FL, USA).

### 2.16. Microglial Morphological Analysis

Furthermore, 63× z-stack images (0.19 μm intervals) of IBA1 stained microglia from treated and untreated mice were collected on a Zeiss LSM confocal microscope (Oberkochen, Germany). Three-dimensional images were then analyzed using the “surfaces” plug-in in Imaris 10.0 to ascertain cell surface area. Individual microglial surface areas were calculated in the advanced statistics tab of Imaris.

### 2.17. Silver Staining

Silver staining was performed using an FD NeuroSilver Kit II (Cat No: PK 301A, FD NeuroTechnologies, INC., Columbia, MD, USA). The procedure was as per the manufacturer’s protocols.

### 2.18. ELISA

The whole brain was dissected out and immersed in liquid nitrogen for 30 s and homogenized using a tissue homogenizer. Tissue homogenates were used for ELISA and Western blot. Brain soluble protein lysates from BK40196-treated and DMSO-treated control mice were used. Specific p-Tau ser396 (Invitrogen, KHB7031), p-Tau (ps199) (Invitrogen, KMB7041) and mast cell protease-6/Mcpt6 (Invitrogen, EM51RB) were performed according to the manufacturer’s protocol on tissue soluble extracts from brain lysates in 1× STEN buffer.

### 2.19. Western Blot

To extract soluble proteins from mouse brain lysates, tissues were isolated and homogenized in 1x STEN buffer (50 mM Tris (pH 7.6), 150 mM NaCl, 2 mM EDTA, 0.2% NP-40, 0.2% BSA, 20 mM PMSF and protease cocktail inhibitor) and centrifuged at 10,000× *g* for 20 min at 4 °C. Extracts were analyzed via Western blot (WB) on 4–12% Criterion™ XT Bis-Tris Protein Gel (Bio-rad, #3450125). Beta-actin (β-actin) was probed (1:3000) with a monoclonal antibody (Emd millipore, MAB1501R). Phospho-c-KIT was probed (1:1000) with a polyclonal antibody (Cat No: 44-496G Invitrogen, Carlsbad, MA, USA). Total tau was probed with a monoclonal antibody (Tau5) (Cat No: AHB0042, Invitrogen, Carlsbad, MA, USA). Phospho-tau was probed with a polyclonal antibody (Ser396) (Cat No: 44-752G, Invitrogen, Carlsbad, MA, USA). Phospho-c-Abl was probed with a polyclonal antibody (Cat No: PA5-114555, Invitrogen, Carlsbad, MA, USA). Phospho-Fyn was probed with a polyclonal antibody (Cat No: PA5-36644, Invitrogen, Carlsbad, MA, USA). WBs were quantified via densitometry using Quantity One 4.6.3 software (Bio Rad, Hercules, CA, USA) and Image J. We performed protein quantification assays and normalized protein levels in the homogenized tissue samples prior to the WB, and also performed ELISA assays. We used an equal amount of proteins for the assays and quantified using standard graphs.

### 2.20. Statistical Analysis

All statistical analysis was performed using GraphPad Prism version 10 (GraphPad software, Inc., San Diego, CA, USA). One-way analysis of variance (ANOVA) (and nonparametric or mixed) tests were used in the comparison of the means of multiple groups. A two-tailed Student’s t test (and nonparametric tests) was used in the comparison of the means of two groups. The Shapiro–Wilk test was used for checking normality before performing statistic tests. Asterisks denote actual *p*-value significances (* <0.05, ** <0.01, *** <0.001 and **** <0.0001), and N is the number of animals or the number of independent experiments (cell culture) per group. Unless otherwise indicated, data are expressed as Mean ± SD.

### 2.21. Ethics and Integrity

All animal work was approved by Georgetown University Animal Care and Use Committee (GUACUC) Protocol# 2016-1194. The work presented in this manuscript conforms with all rigorous and ethical standards for research conduct, data collection and analysis and data dissemination.

## 3. Results

### 3.1. Screening of Kinase Binding of BK40196

**Figure 1:**
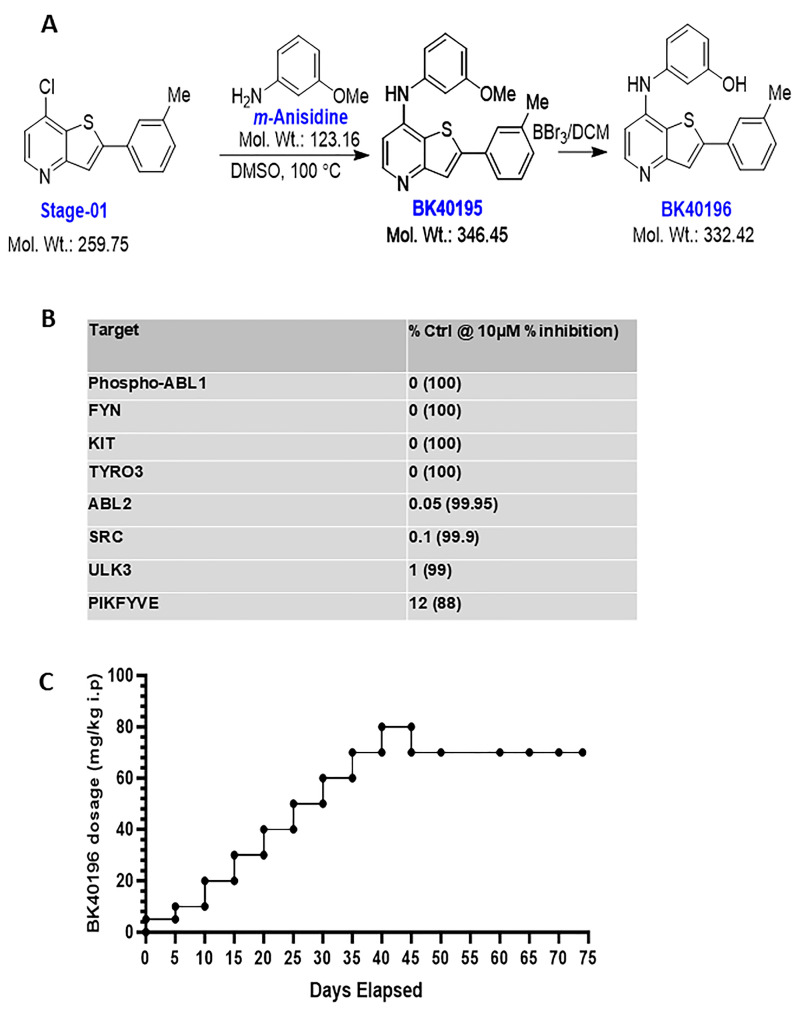
BK40196 can cross BBB, inhibit c-KIT and enhance autophagy. (**A**) Schematic of chemical structure of BK40196 and its synthesis. (**B**) BK40196 inhibits c-KIT with the competitive binding assay values for some selected kinases in vitro. (**C**) Administration of BK40196 in WT mice revealed the maximum tolerable dose in vivo.

The binding interactions between BK40196 (Figure 1A) against 450 human kinases and disease-relevant mutant variants were quantitatively measured using KINOMEscan (Figure 1B) and showed that the POC value with zero corresponded with BK40196 completely abolishing ligand binding (100% inhibition) of human c-KIT, ABL1-phosphorated, FYN and TYRO3. The lowest range of POC 0 ≤ x < 0.1 showed that BK40196 strongly prevents multiple kinases binding associated with neurodegeneration and neuroinflammation; these targets include SRC (POC 0.1 and 99.9% of inhibition). Other kinases which BK40196 targets include PIKFYVE (POC 12 and 88% of inhibition). BK40196 also prevents autophagy-related proteins ULK3 (POC 1.0 and 99% of inhibition).

### 3.2. Pharmacokinetics and Maximal Tolerated Dose

**Figure 2:**
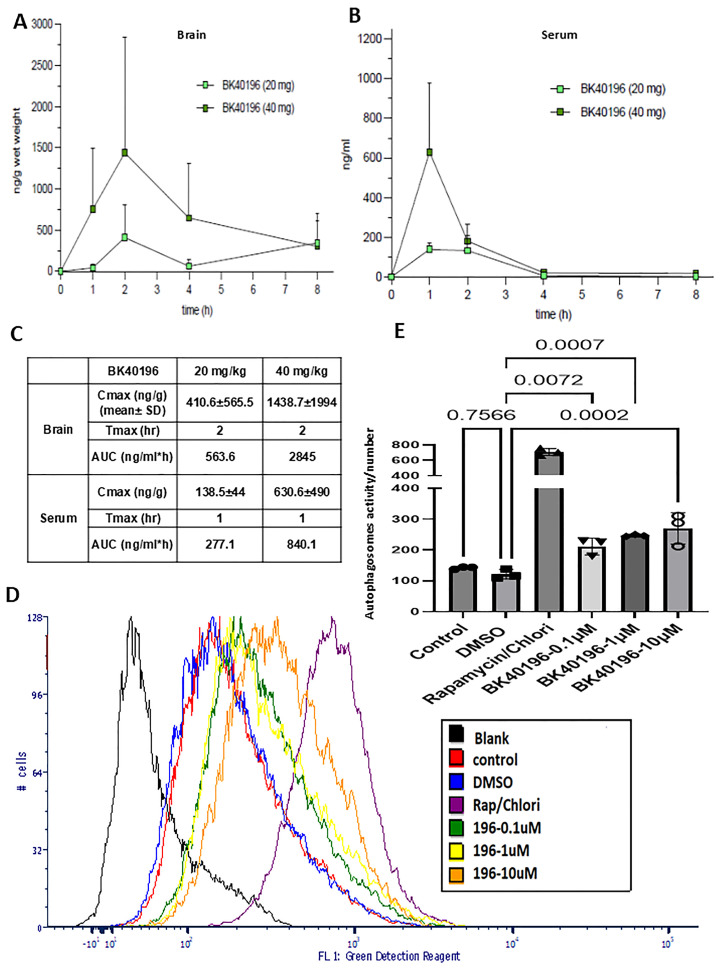
(**A**) Pharmacokinetic analysis revealed brain penetrance of BK40196 at multiple doses compared to (**B**) blood (serum) analysis. (**C**) Pharmacologic parameters of BK40196 in mice. (**D**,**E**) Flow cytometric analysis of SH-SY5Y cells revealed increased production of autophagic vacuoles in cells treated with BK40196.

We then determined the maximum tolerated dose by conducting dose escalation study in 6–8-month-old wild-type C57BL/6N mice. A dose escalation study (Figure 1C) showed no visible adverse effects in mice treated with BK40196 till 80 mg/kg i.p daily with one female mouse dying (autopsy showed pulmonary congestion and liver necrosis); then, we reduced the dosage to 70 mg/kg i.p daily. There was no other mortality or adverse effects noticed. Therefore, 70 mg/kg i.p may be near to or the maximum tolerated dose in mice. To determine if BK40196 can cross the blood–brain barrier (BBB), we performed pharmacokinetics (PKs). BK40196 was injected into male and female C57BL/6 mice (5–6 months old) at a single dosage of 20 or 40 mg/kg i.p. PK analysis using liquid chromatography–mass spectrometry showed that BK40196 was brain penetrant (Figure 2A,B,C) and reached peak concentration in the serum at 1 h and in the brain at 2 h following the injections. The serum–brain ratio of BK40196 was greater than 30%, indicating that this molecule readily enters the CNS.

### 3.3. BK40196 Induces Autophagy In Vitro

KINOMEscan showed that BK40196 has high affinity to autophagy-associated kinases such as ULK3 (POC 1.0) and PIKFYVE (POC 12). To establish that treatment with BK40196-induced autophagy, we treated human neuroblastoma SH-SY5Y cells with rapamycin/chloroquine as a positive control, DMSO as a negative control, or an ascending dose (0.1μM, 1μM or 10μM) of BK40196 for 8 h. We found that treatment with BK40196 (Figure 2E,D) resulted in significant dose-dependent increases in production of autophagic vacuoles, indicating induction of autophagy in cells.

### 3.4. BK40196 Inhibits c-KIT, Phospho-c-Abl and Phospho-Fyn in rTg4510 Mice

**Figure 3:**
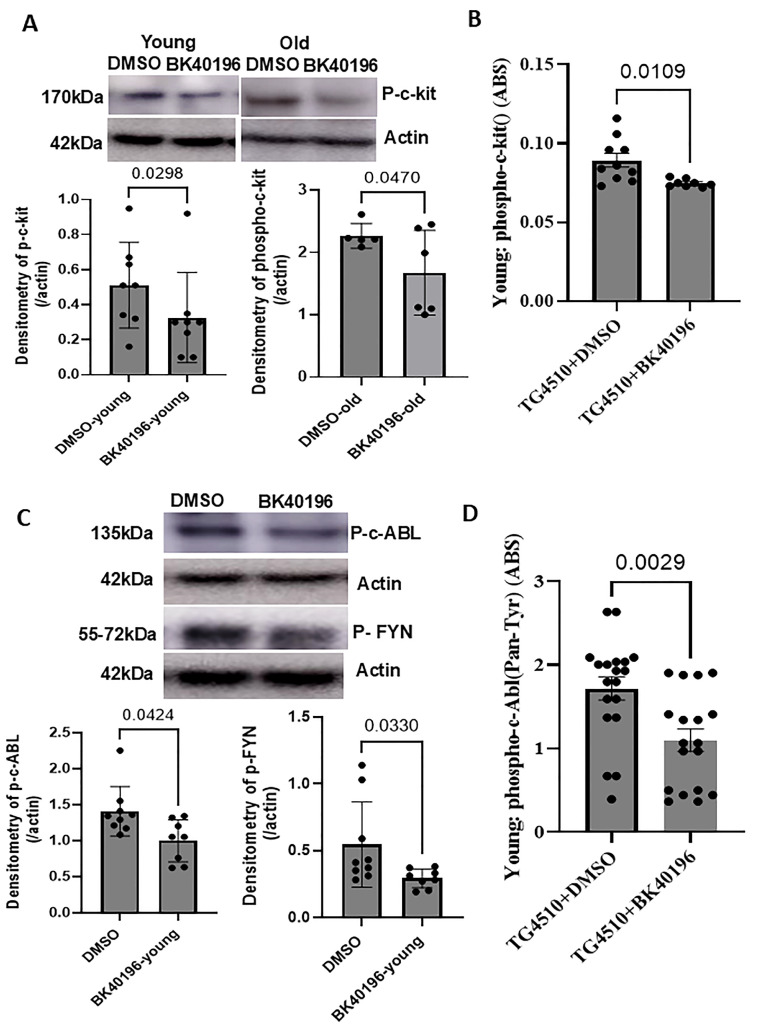
BK40196 inhibits c-KIT, phospho-c-Abl and phosphor-Fyn in Tg4510 mice. Young (5–6 months) and old (11–12-month-old) Tg4510 mice were treated with BK4096 at a daily dosage of 20 mg/kg i.p for 3–5 weeks. WB analysis of brain lysates showed that BK40196 significantly reduced c-KIT levels (**A**), phospho-c-Abl and phosphor-Fyn levels (**C**) in Tg4510 mice. ELISA for c-KIT (**B**) and phosphor-c-Abl (**D**) in Tg4510 mice revealed a decrease in protein levels compared to the vehicle-treated controls. N = 8–10, two-tailed t test was used for statistics and *p* values were shown in the graphs.

We verified the screening results that BK40196 is a c-KIT inhibitor as shown in KINOMEscan. Activation of c-KIT leads to its auto-phosphorylation and leads to APP phosphorylation and Aβ production. To test if BK40196 inhibits c-KIT in vivo, we used male and female young (5–6-month-old) and old (11–12-month-old) rTg4510 mice and treated them with BK4096 at a daily dosage of 20 mg/kg i.p for 3–5 weeks. Western blot analysis of brain lysates showed that BK40196 significantly reduced c-KIT levels compared with vehicle (DMSO) control (Figure 3A) in both young and old rTg4510 mice, and the results were confirmed by ELISA (Figure 3B).

Similarly, KINOMEscan showed that BK40196 completely abolished ligand binding of human phospho-ABL1 and FYN. Western blot analysis of brain lysates showed that BK40196 significantly reduced phospho-c-ABL and phospho-FYN levels compared with vehicle (DMSO) control (Figure 3C) in young rTg4510 mice, and the results were confirmed by ELISA (Figure 3D), indicating that BK40196 inhibits c-ABL in rTg4510 mice. Taken together, these data suggest that BK40196 is a multi-kinase inhibitor of c-ABL/FYN/c-KIT.

### 3.5. BK40196 Prevents Mast Cell Proliferation and Maturation In Vitro

**Figure 4:**
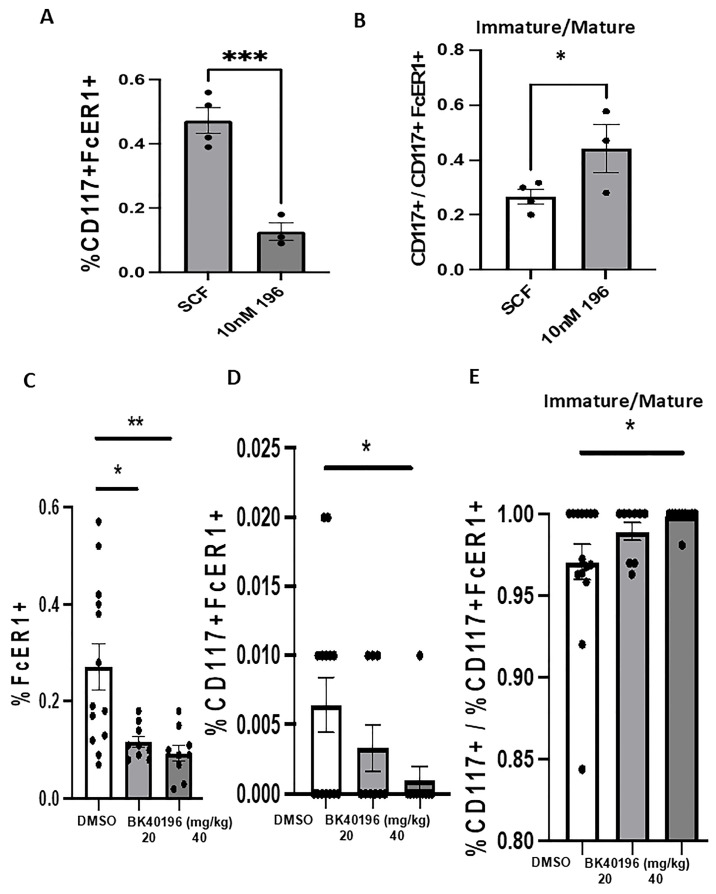
BK40196 prevents mast cell proliferation and maturation in vitro *and* in vivo. Flow cytometric analysis of the LUVA cells treated with BK40196 revealed lower numbers of CD117+/FcER1+ cells (**A**) and a greater ratio of c-KIT (CD117)+ labeled cells to CD117+/FcεR1+ labeled cells (**B**) compared to SCF-stimulated cells. TgAPP mice treated with 20 mg/kg or 40 mg/kg of BK40196 or DMSO i.p for 3 weeks. Flow cytometric analysis of the mast cells isolated from the spleen of TgAPP mice showed that BK40196 treatment reduced the number of FcER1+ cells (**C**) and CD117+/FcER1+ cells (**D**) and enhanced the ratio of c-KIT (CD117)+ labeled cells to CD117+/FcεR1+ labeled cells (**E**) compared to the DMSO controls. ELISA analysis of brain lysates from Tg4510 mice treated with BK40196 at 20 mg/kg i.p for 5 weeks revealed decreased beta-tryptase levels compared to DMSO controls. * *p* < 0.05, ** *p* < 0.01, *** *p* < 0.00, Ordinary one-way ANOVA or two tailed student t test. All values presented as Mean ± SD.

c-KIT is a receptor TK and the activation by its cytokine ligand, stem cell factor (SCF), phosphorylates multiple intracellular proteins including proteins which are responsible for mast cell function. The over-reacting FceR1-mediated degranulation of mast cells release large amount of mediators, like tryptase, which may damage the brain. Mast cells are tissue-resident cells of hematopoietic lineage which are derived from CD13 + CD34 + KIT(CD117)+ bone marrow progenitors and develop from CD34+/CD117+ pluripotent progenitor cells originating in the bone marrow. The progression of these cells to fully mature mast cells is dependent on c-KIT activation which occurs because of SCF-induced c-KIT auto-phosphorylation. To determine whether BK40196 inhibits c-KIT and mast cell maturation in vitro, human mast cells (LUVA cells), which express functional c-KIT, were cultured and treated with SCF, the endogenous ligand of c-KIT (CD117), before being treated with BK40196 and harvested for flow cytometry analysis. Cells were labeled with PE anti-c-KIT to isolate c-KIT positive mast cell progenitors and APC/fire anti-FcεR1α to identify mature mast cells. LUVA cells exposed to BK40196 (Figure 4A) displayed significantly lower numbers of double labeled (CD117+/FcεR1+) cells than cells treated with SCF, indicating BK40196 inhibition of the proliferation of mast cells. Cells treated with BK40196 showed more increased ratios of immature mast cells than the LUVA cells treated with SCF (Figure 4B), indicating a blockade of cell maturation. This suggests that inhibition of c-KIT with BK40196 is sufficient to prevent maturation and proliferation of LUVA cells in vitro.

### 3.6. BK40196 Inhibits Peripheral Mast Cell Maturation In Vivo

To investigate how inhibition of c-KIT with BK40196 affects peripheral mast cell populations in vivo, we collected the spleen from TgAPP mice treated with 20 mg/kg or 40 mg/kg of BK40196 or DMSO i.p for 3 weeks and isolated mast cells for analysis via flow cytometry. The spleen was homogenized and sorted for mast cells using a triple gating strategy: 647 anti-CD45 to isolate leukocytes, PE anti-c-KIT(CD117+) to isolate mast cell progenitors, and APC/fire anti-FcεR1α to identify mature mast cells. The ratio of immature to mature mast cells (c-KIT+ vs. c-KIT+/ FcεR1α+) was compared between c-KIT inhibited and DMSO-treated controls to determine the functional consequence of c-KIT inhibition on peripheral mast cell maturation. We found that mice treated with 20 and 40 mg/kg BK40146 (Figure 4C,D) displayed a significantly lower number of both single labeled (FcεR1α+) and double labeled (CD117+/ FcεR1α+) cells than the mice treated with DMSO, indicating the inhibition of mast cell proliferation and expression of FcεR1. We also found that mice treated with 40 mg/kg BK40146 (Figure 4E) exhibited a significantly greater ratio of immature to mature mast cells when compared to DMSO-treated mice, whereas 20 mg/kg of BK40196-treated mice displayed a similar, albeit not statistically significant, trend (Figure 4E), indicating that c-KIT inhibition with BK40196 prevents maturation of peripheral mast cell progenitors.

### 3.7. BK40196 Reduces Mast Cell-Derived Tryptase in the Brain of rTg4510 Mice

**Figure 5:**
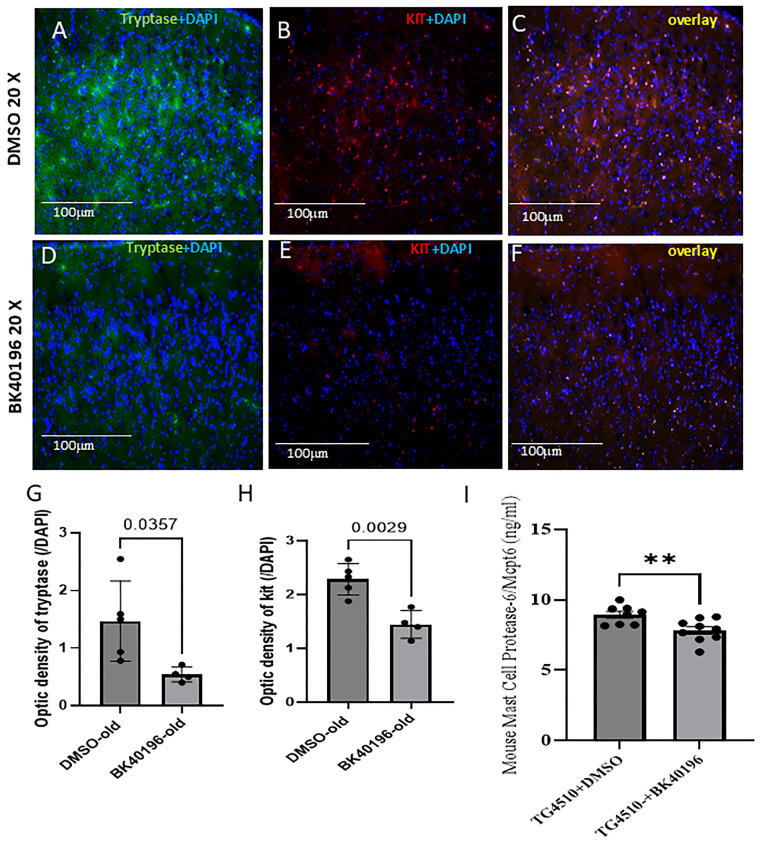
BK40196 reduces mast cell neuroinflammation in Tg4510 mice. Tg4510 mice (11–12 months old) were treated with an intraperitoneal injection of vehicle or BK40196 at the daily dosage of 20 mg/Kg for 3 weeks. Co-staining of mast cell marker tryptase and DAPI in 20 µm thick mouse brain sections show that BK40196 decreased staining of cortical tryptase (**D**) compared to the DMSO-treated brain (**A**). Co-staining of c-KIT and DAPI showing that BK40196 decreased staining of cortical c-KIT (**E**) compared to the DMSO-treated brain (**B**) in Tg4510 mice. The overlay of tryptase and c-KIT interaction showing in DMSO- (**C**) and BK40196-treated (**F**) mice. Scale bars, 200 μm. Quantification of tryptase (**G**) and c-KIT (**H**). ELISA analysis of brain lysates from Tg4510 mice treated with BK40196 at 20 mg/kg i.p for 5 weeks revealed decreased beta-tryptase levels compared to the DMSO controls (**I**). Two-tailed Student’s t test was used for analysis. N = 3–5 mice per group. All values presented as Mean ± SD. ** *p* < 0.01 two tailed student *t* test.

We stained tryptase, the most abundant granule-derived serine proteinase contained in mast cells and a pro-inflammatory marker secreted by activated mast cells. We observed that BK40196 treatment significantly reduced c-Kit and tryptase staining (Figure 5D–H) in the cortex of 10–12-month-old rTg4510 mice compared to the DMSO group (Figure 5A–C,G,H), indicating that BK40196 inhibits c-KIT and prevents tryptase release by activated mast cells in the brain. We further confirmed tryptase reduction in the brain of our mice by performing ELISA for mast cell protease-6/Mcpt6 on lysates from BK40196 treated vs. control rTg4510 mice. We observed a significant decrease in tryptase levels in BK40196-treated (20 mg/kg i.p) mice (Figure 5I), indicating BK40196 inhibition of peripheral mast cell maturation and prevention of central mast cell activation, hence BK40196 anti-neuroinflammatory effects. Please note that antibodies to directly detect mast cells in the brain are not available.

### 3.8. BK40196 Inhibits c-KIT and Reduces Microglial Activity

**Figure 6:**
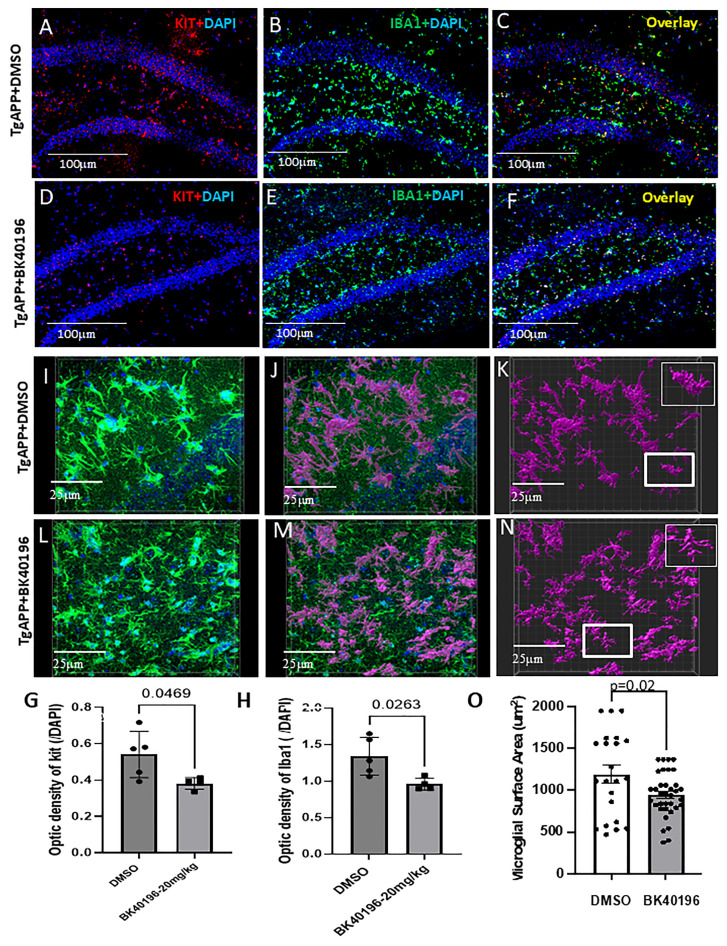
BK40196 inhibit c-KIT and reduced microglial neuroinflammation in TgAPP mice. TgAPP mice were treated with an intraperitoneal injection of vehicle or BK40196 at the daily dosage of 20 mg/Kg for 3 weeks. Co-staining of c-KIT and DAPI in 20 µm thick mouse brain sections showing that BK40196 (**D**) decreased staining of hippocampal c-KIT compared to the DMSO-treated brain (**A**). Co-staining of IBA1 and DAPI showing BK40196 decreased staining of hippocampal IBA1 (**E**) compared to the DMSO-treated brain (**B**). The overlay of c-KIT and IBA1 interaction showing in DMSO- (**C**) and BK40196-treated (**F**) mice. Scale bars, 200 μm. Quantification of c-KIT (**G**) and IBA1 (**H**). IMARIS 3D reconstruction and analysis revealed microglia from DMSO-treated mice (**I**,**J**,**K**) displayed significantly greater surface area than microglia from mice treated with BK40196 (**L**,**M**,**N**), verified by the quantification (**O**). Individual microglia from DMSO-treated mice (**K**, Insert) displayed ameboid shape and more ramified microglia were detected in mice treated with BK40196 (**N**, Insert). Two-tailed Student’s t test was used for analysis. All values presented as Mean ± SD.

Neuroinflammation is the main culprit of most neurodegenerative diseases. Brain mast cells and microglia cells are two main important resident types of immune cells in the CNS. They respond to many CNS perturbations including beta-amyloid plaques and neurofibrillary tangles. However, the failure to resolve the pathogenic process can lead to persistent activation, release of pro-inflammatory mediators (e.g., tryptase) and neuroinflammation. To examine the effects of BK40196 on microglial activation and neuroinflammation in vivo, we performed immunohistochemistry and morphological analysis of microglia from the brains of treated and untreated TgAPP mice. We stained for IBA1 (ionized calcium binding adaptor molecule 1), a microglia/macrophage-specific calcium binding protein to identify microglia in the brain. We found that treatment with BK40196 significantly inhibited c-KIT expression (Figure 6A,D,G) and reduced IBA1 staining (Figure 6B,E,H) levels compared to the DMSO group in the hippocampus of TgAPP mice.

To further investigate the morphology of activated microglia, we used “surfaces” plug-in Imaris to analyze microglia. We observed that the branches of microglia cells are short and stubby, and the cell bodies appear amoeboid in the DMSO-treated brain (Figure 6I–K), indicating microglia activation. Treatment with BK40196 transformed the microglia to normal/inactivate phenotype (Figure 6L–N). Quantitation of microglial surface area showed that BK40196 significantly reduced microglia activation (Figure 6O), suggesting anti-neuroinflammatory effects of BK40196 in TgAPP mice.

### 3.9. BK40196 Improves Motor Performance and Cognitive/Memory Behavior in rTg4510 Mice

**Figure 7:**
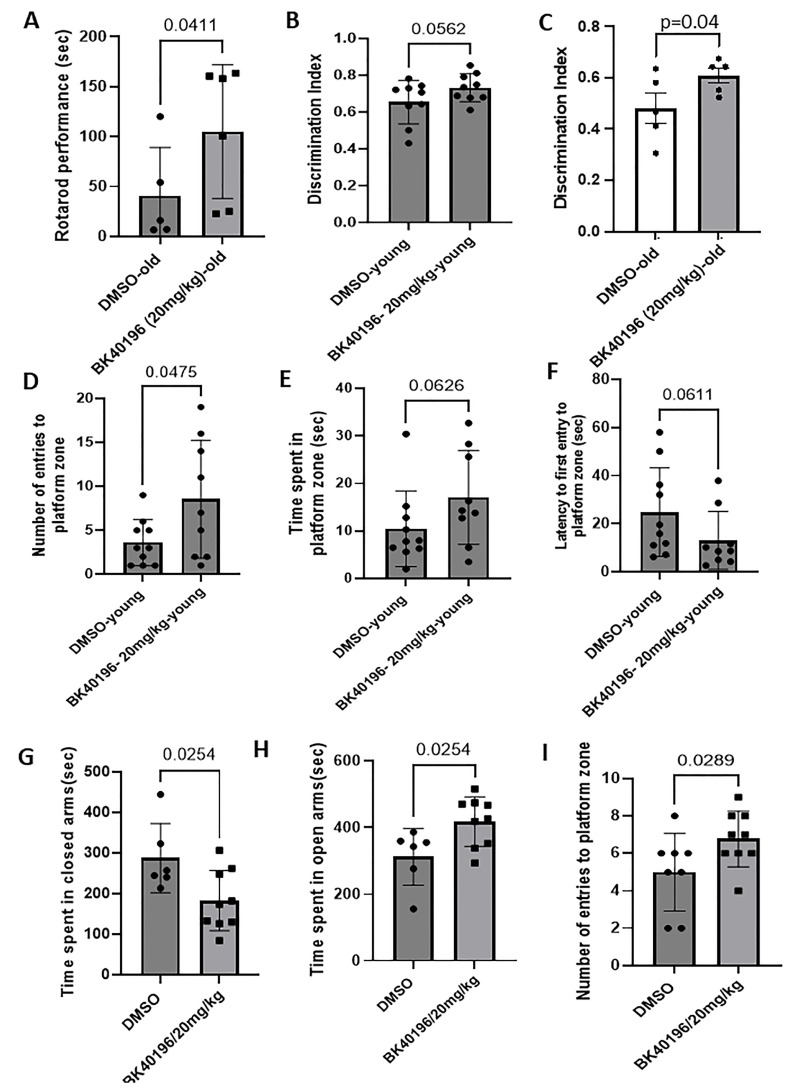
BK40196 improved motor performance and cognitive behavior in Tg4510 and TgAPP mice. Young (5–6 months) and old (11–12-month-old) Tg4510 mice were treated with BK4096 at a daily dosage of 20 mg/kg i.p for 3–5 weeks. The rotarod test showed that BK40196 significantly improved the motor performance (**A**). The NOR test showed that BK40196 significantly improved the cognitive memory in Tg4510 mice (**B**,**C**). The Morris water maze test showed that BK40196 treatment dramatically increased the number of entries to the platform located zone and time spent in that zone and decreased the latency to first entry to the platform located zone in Tg4510 mice (**D**–**F**). TgAPP mice treated with 20 mg/kg of BK40196 or DMSO i.p for 3 weeks. The elevated plus maze test revealed that BK40196 treatment significantly reduced time spent in closed arms and increased the time spent in open arms (**G**,**H**), and the Morris water maze test showed that BK40196 treatment dramatically increased number of entries to the platform located zone compared to DMSO-treated TgAPP mice (**I**).

We used rTg4510 (11–12 months old) mice with an overexpression of mutant human Tau and AD/FTD-associated mutations. We treated these mice with BK40196 at a daily i.p. dosage of 20 mg/kg or DMSO (vehicle) for 3 consecutive weeks before performing behavioral analysis. The rotarod test was performed and showed that BK40196 significantly improved the motor performance in rTg4510 mice compared to DMSO control mice (Figure 7A). To test cognition, a novel object recognition test was performed in young (5–6 months old) and old (11–12 months old) male and female rTg4510 mice with BK40196 at a daily intraperitoneal dosage of 20 mg/kg or DMSO (vehicle) for a consecutive 3–5 weeks. BK40196 treatment showed an age-dependent improvement of memory in rTg4510 mice compared to vehicle-treated mice (Figure 7B,C) and BK40196 significantly increased the time spent in exploring the novel object than the DMSO-treated controls. To further investigate spatial learning and memory, we performed the Morris water maze test and found that mice treated with BK40196 spent a significantly longer time in platform zone and made more frequent entries/visits to that zone compared to vehicle control mice (Figure 7D,E). Also, the BK40196-treated mice needed less time identifying the platform zone compared to vehicle control mice in the Morris water test (Figure 7F).

### 3.10. BK40196 Improves Cognitive/Memory Behavior and Alleviates Anxiety in TgAPP Mice

To further evaluate BK40196 on anxiety, we treated TgAPP (16–18 months old) with BK40196 at a daily i.p. dosage of 20 mg/kg or DMSO (vehicle) for three consecutive weeks before performing behavioral analysis. The elevated plus maze test, a measure of anxiety-like behavior based on the general aversion of rodents to open spaces, showed that TgAPP mice treated with BK40196 significantly spent less time in closed arms (Figure 7G) and more time in open arms (Figure 7H) compared to DMSO-treated control mice, indicating anti-anxiety effects of BK40196 in TgAPP mice. The Morris water maze also showed that TgAPP mice treated with BK40196 significantly increased the number of entries to the platform located zone compared to vehicle-treated TgAPP mice (Figure 7I), indicating improved spatial learning and memory.

### 3.11. BK40196 Reduces Ptau and Beta-Amyloid Levels and Prevents Cell Death

**Figure 8:**
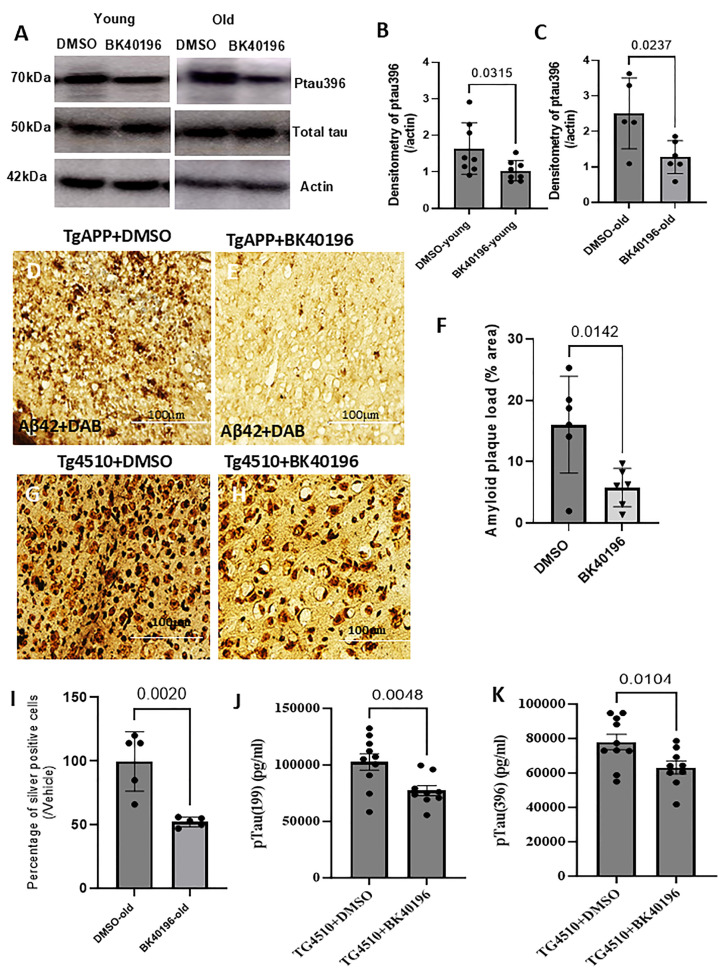
BK40196 facilitated the clearance of ptau and beta-amyloid and prevented cell death in mouse models of AD. Young and old Tg4510 mice were treated with an intraperitoneal injection of vehicle or BK40196 at the daily dosage of 20 mg/Kg for 3–5 weeks. WB of brain lysates showing ptau396 and total tau levels relative to actin on 4–12% NuPAGE SDS gel (**A**) and the densitometries (**B**,**C**). ELISA measuring levels of ptau199 (**J**) and ptau396 (**K**). Immunohistochemistry staining of 20 µm thick mouse brain sections showing beta-amyloid+ neurons co-labeled with Nissl staining in the ventral DG of hippocampus (**D**,**E**), verified by quantification of optic density (**F**). Silver staining of brain sections showing the positive cells in cortex (**G**,**H**), quantified by silver positive cell counting (**I**). Statistical analysis was performed by two-tailed Student’s *t* test. N = 4–8 mice per group. All values presented as Mean ± SD.

To evaluate if BK40196 can promote the clearance of phosphorylated tau, a major component of neurofibrillary tangles, we performed WB analysis on the brain lysates harvested from above young (5–6-month-old) and old (11–12-month-old) rTg4510 mice. The results showed that treatment with BK40196 significantly reduced phosphorylated ptau396 levels in both young and old rTg4510 mice (Figure 8A–C) and had no effect on total Tau levels (Figure 8A).

To investigate the effects of BK40196 on beta-amyloid levels in neurodegeneration, we performed DAB staining of brain sections in TgAPP mice. We found that mice treated with BK40196 showed a significant reduction in beta-amyloid levels in ventral hippocampus region compared to that in control mice treated with the DMSO vehicle (Figure 8D–F). Ventral hippocampus is a major brain structure associated with anxiety/stress/emotion, which is in good accordance with our behavioral results of anti-anxiety effects of BK40196.

To evaluate the neuroprotective effects of BK40196, we performed cupric silver staining in 20 µm thick brain sections of rTg4510 mice. We found that BK40196 treatment mice showed a significantly lower number of stained cells in the cortex of rTg4510 mice (Figure 8G–I) compared to DMSO treatment brains, suggesting BK40196 reduces tau hyper-phosphorylation and prevents cell loss in neurodegeneration. ELISA further confirmed that BK40196 treatment resulted in significantly reduced levels of phosphorylated ptau199 and ptau396 levels (Figure 8J,K) compared to vehicle-treated rTg4510 mice.

## 4. Discussion

We synthesized a novel small molecule, BK40196, that can readily penetrate the brain and target both peripheral immunity via mast cells and CNS pathology of neurodegeneration via mitigation of microglial activity. BK40196 is a unique first-in-class small molecule compared to existing FDA-approved TKIs that very poorly penetrate the brain and carry specific safety and precautions warnings, including myelosuppression and QTc prolongation. BK40196 also displays synergistic multi-kinase targets, including key tyrosine kinases, c-Abl, KIT, Fyn and non-tyrosine kinases such as ULK3 and PYKEFIVE that share common mechanisms to modulate autophagy and inflammation. BK40196 may also be able to target both central as well as peripheral mast cells’ maturation and release of pro-inflammatory tryptase, through selective c-KIT inhibition, suggesting a high potential of success as a therapeutic agent to alleviate inflammatory diseases. We identified the specific kinases that BK40196 targets from over 450 candidates and the potential binding to kinases that are primarily involved with disease pathogenesis: c-Abl, c-KIT and Src-kinase FYN, which are associated with autophagy, microglial activation and tau phosphorylation, and have been investigated as potential therapeutic targets [11,15,16]. BK40196 is an inhibitor of c-Abl, which makes it a strategic therapeutic agent for idiopathic PD due to the widely established effects of c-Abl on alpha-synuclein [17,18] degradation via autophagy [19,20]. Src regulates secretase activation and tau truncation and phosphorylation [21] and the Src/c-Abl pathway was demonstrated to prolong neuronal survival in models of ALS-iPSC-derived motor neurons [22]. C-Abl is a critical sensor of oxidative stress via the c-Abl-p38 alpha signaling pathway [23]. The Src kinase Fyn is also primarily targeted by BK40196 and the role of Fyn has long been established in aggravating tau phosphorylation and amyloid toxicity and causing synaptic and dendritic deficits in AD and the tauopathies [24,25,26]. Fyn inhibition has been promoted as a therapeutic strategy [25,27,28,29], and upregulation of Fyn expression is associated with microglial activity and tau pathology in AD and DLB [30]. In a systematic comparison of various tyrosine kinase inhibitors, we demonstrated that multi-kinase inhibition is optimal in neurodegeneration, perhaps due to simultaneously congruent mechanisms of autophagy, inflammation and oxidative stress [3]. Our data indicate that treatment with BK40196 induces autophagy in SH-SY5Y cells and reduce β-amyloid (Aβ) and various epitopes of hyper-phosphorylated tau in rTg4510. BK40196 significantly reduces microglial activity and prevents cell death suggesting an anti-inflammatory role. Therefore, kinase inhibition provides a golden opportunity to investigate the differential effects of BK40196 in AD and the tauopathies [25,31]. Mastinib is an example of a c-KIT inhibitor that is being investigated as a potential therapy in AD [12,32,33] and multiple sclerosis [13,34,35,36]. BK40196 targets c-KIT and the bone marrow-derived mast cells that are key to innate immunity. Committed bone marrow mast cell progenitors are released into the bloodstream and migrate to peripheral tissues (e.g., spleen) where they mature [37]. c-KIT activation induces mast cell maturation and function, leading to the release of pro-inflammatory factors, including tryptase [38]. Inhibition of c-KIT via BK40196 affects maturation and activation of mast cells peripherally and may also regulate their cross-talk with CNS microglia as our data suggest as tryptase is significantly reduced in the brain in association with reduced microglial activation and reduction in amyloid and p-tau. BK40196 may also directly inhibit c-KIT activity in the brain, therefore affecting microglial activity and neuroinflammation independent of the peripheral effects of mast cells. The function of mast cells in peripheral inflammatory conditions is well recognized and increasing evidence indicates that in the brain, inflammation is a driving force of neurodegeneration [39,40,41,42]. Mast cells and microglia may interact to produce immune responses in AD and PD [43], suggesting mast cells as neuroprotective and anti-inflammatory mediators [44]. Mast cells are increased in PD models [45,46] and microglia and mast cell activation are implicated in MPTP models of PD [47]. Inflammation in peripheral motor axon degeneration in amyotrophic lateral sclerosis (ALS) is associated with mast cells’ activation [48] and c-KIT inhibition abrogates TDP43 pathology in ALS [49]. Therefore, c-KIT inhibition may either lead to orchestration of microglia–mast cells interaction and tryptase release, thus mediating a neuroimmune-mast cell axis, or it may peripherally tame mast cell activation and prevent the release of pro-inflammatory factors that can enter the CNS.

BK40196 binds to other protein kinases, including *FYVE* finger-containing phosphoinositide *kinase* (PYKEFIVE) and Unc51-like kinase (ULK)3, which induce autophagic clearance of toxic proteins [50,51,52,53], in agreement with our data showing amyloid and pTau clearance in association with behavioral improvement. PIKFYVE inhibition has been shown to induce autophagy [52,54,55]. Autophagy prevents accumulation of p-tau and Aβ and reduces plaque deposits [56,57] in several models of AD [58,59,60] and the tauopathies [3,10,11]. However, more work is required to determine the effects of BK40196 on PYKFIVE and ULK3 and this is an ongoing area of investigation in our lab that is beyond the scope of this manuscript.

In summary, we developed a brain-penetrant small molecule that can potentially induce congruent pathways that are putatively involved in overlapping functions of autophagy, inflammation and oxidative stress. BK40196 may affect peripheral immunity via mast cells and central microglial activity either directly via direct c-KIT inhibition or in unison via peripheral and central modulation of the inflammatory response. Furthermore, BK40196 induces autophagy and clearance of amyloids suggesting harmonious mechanisms that favor concurrent autophagy and anti-inflammation.

**Limitations**: Considering the historical side effects of tyrosine kinase inhibition, BK40196 must be further studied to investigate its effects on cardiovascular and myeloid-toxicity in future toxicology studies. Taken together, BK40196 is a candidate for the treatment of neurodegenerative diseases, including tau pathology in AD and frontotemporal dementia (FTD).

## Data Availability

The data supporting the current study are available from the corresponding author on request.

## Abbreviations

AD = Alzheimer’s disease, ALS = amyotrophic lateral sclerosis, Aβ = amyloid-beta, APP = amyloid precursor protein, BBB = blood–brain barrier, DMSO = dimethyl sulfoxide, DAB = 3, 3′ diaminobenzidine, FTD = Fronto-Temporal Dementia, IP = intraperitoneal, PD = Parkinson’s disease, pTau = phosphorylated tau, PK = pharmacokinetic, TK = tyrosine kinase.

## References

[1] Jiang W., Ji M. (2019). Receptor tyrosine kinases in PI3K signaling: The therapeutic targets in cancer. Semin. Cancer Biol..

[2] Hu J., Zhang D., Tian K., Ren C., Li H., Lin C., Huang X., Liu J., Mao W., Zhang J. (2023). Small-molecule LRRK2 inhibitors for PD therapy: Current achievements and future perspectives. Eur. J. Med. Chem..

[3] Fowler A.J., Hebron M., Missner A.A., Wang R., Gao X., Kurd-Misto B.T., Liu X., Moussa C.E. (2019). Multikinase Abl/DDR/Src Inhibition Produces Optimal Effects for Tyrosine Kinase Inhibition in Neurodegeneration. Drugs R D.

[4] Hebron M., Peyton M., Liu X., Gao X., Wang R., Lonskaya I., Moussa C.E. (2017). Discoidin domain receptor inhibition reduces neuropathology and attenuates inflammation in neurodegeneration models. J. Neuroimmunol..

[5] Tang H., Sun Y., Fachim H.A., Cheung T.K.D., Reynolds G.P., Harte M.K. (2023). Elevated Expression of Two Pore Potassium Channel THIK-1 in Alzheimer’s Disease: An Inflammatory Mechanism. J. Alzheimers Dis..

[6] Fraser J., Cabodevilla A.G., Simpson J., Gammoh N. (2017). Interplay of autophagy, receptor tyrosine kinase signalling and endocytic trafficking. Essays Biochem..

[7] Lemmon M.A., Schlessinger J. (2010). Cell signaling by receptor tyrosine kinases. Cell.

[8] Guglietti B., Sivasankar S., Mustafa S., Corrigan F., Collins-Praino L.E. (2021). Fyn Kinase Activity and Its Role in Neurodegenerative Disease Pathology: A Potential Universal Target?. Mol. Neurobiol..

[9] Stevenson M., Varghese R., Hebron M.L., Liu X., Ratliff N., Smith A., Turner R.S., Moussa C. (2023). Inhibition of discoidin domain receptor (DDR)-1 with nilotinib alters CSF miRNAs and is associated with reduced inflammation and vascular fibrosis in Alzheimer’s disease. J. Neuroinflammation.

[10] Lonskaya I., Hebron M.L., Selby S.T., Turner R.S., Moussa C.E. (2015). Nilotinib and bosutinib modulate pre-plaque alterations of blood immune markers and neuro-inflammation in Alzheimer’s disease models. Neuroscience.

[11] Mahul-Mellier A.L., Fauvet B., Gysbers A., Dikiy I., Oueslati A., Georgeon S., Lamontanara A.J., Bisquertt A., Eliezer D., Masliah E. (2014). c-Abl phosphorylates alpha-synuclein and regulates its degradation: Implication for alpha-synuclein clearance and contribution to the pathogenesis of Parkinson’s disease. Hum. Mol. Genet..

[12] Folch J., Petrov D., Ettcheto M., Pedros I., Abad S., Beas-Zarate C., Lazarowski A., Marin M., Olloquequi J., Auladell C. (2015). Masitinib for the treatment of mild to moderate Alzheimer’s disease. Expert Rev. Neurother..

[13] Arsenault S., Benoit R.Y., Clift F., Moore C.S. (2022). Does the use of the Bruton Tyrosine Kinase inhibitors and the c-kit inhibitor masitinib result in clinically significant outcomes among patients with various forms of multiple sclerosis?. Mult. Scler. Relat. Dis..

[14] Stevenson M., Hebron M.L., Liu X., Balaraman K., Wolf C., Moussa C. (2024). c-KIT inhibitors reduce pathology and improve behavior in the Tg(SwDI) model of Alzheimer’s disease. Life Sci. Alliance.

[15] Dhawan G., Combs C.K. (2012). Inhibition of Src kinase activity attenuates amyloid associated microgliosis in a murine model of Alzheimer’s disease. J. Neuroinflammation.

[16] Nygaard H.B., van Dyck C.H., Strittmatter S.M. (2014). Fyn kinase inhibition as a novel therapy for Alzheimer’s disease. Alzheimers Res. Ther..

[17] Crunkhorn S. (2023). Suppressing c-Abl in Parkinson disease. Nat. Rev. Drug Discov..

[18] Werner M.H., Olanow C.W. (2022). Parkinson’s Disease Modification through Abl Kinase Inhibition: An Opportunity. Mov. Disord..

[19] Hebron M.L., Lonskaya I., Moussa C.E. (2013). Nilotinib reverses loss of dopamine neurons and improves motor behavior via autophagic degradation of alpha-synuclein in Parkinson’s disease models. Hum. Mol. Genet..

[20] Karim M.R., Liao E.E., Kim J., Meints J., Martinez H.M., Pletnikova O., Troncoso J.C., Lee M.H.K. (2020). α-Synucleinopathy associated c-Abl activation causes p53-dependent autophagy impairment. Mol. Neurodegener..

[21] Jiang Y., Li L., Wu R., Wu L., Zhang B., Wang J.Z., Liu R., Liu F., Wang J., Wang X. (2023). c-Src regulates delta-secretase activation and truncated Tau production by phosphorylating the E3 ligase Traf6. J. Biol. Chem..

[22] Imamura K., Izumi Y., Watanabe A., Tsukita K., Woltjen K., Yamamoto T., Hotta A., Kondo T., Kitaoka S., Ohta A. (2017). The Src/c-Abl pathway is a potential therapeutic target in amyotrophic lateral sclerosis. Sci. Transl. Med..

[23] Wu R., Chen H., Ma J., He Q., Huang Q., Liu Q., Li M., Yuan Z. (2016). c-Abl-p38α signaling plays an important role in MPTP-induced neuronal death. Cell Death Differ..

[24] Ittner L.M., Ke Y.D., Delerue F., Bi M.A., Gladbach A., van Eersel J., Wölfing H., Chieng B.C., Christie M.J., Napier I.A. (2010). Dendritic Function of Tau Mediates Amyloid-β Toxicity in Alzheimer’s Disease Mouse Models. Cell.

[25] Yang K., Belrose J., Trepanier C.H., Lei G., Jackson M.F., MacDonald J.F. (2011). Fyn, a Potential Target for Alzheimer’s Disease. J. Alzheimers Dis..

[26] Cochran J.N., Hall A.M., Roberson E.D. (2014). The dendritic hypothesis for Alzheimer’s disease pathophysiology. Brain Res. Bull..

[27] Smith L.M., Zhu R., Strittmatter S.M. (2018). Disease-modifying benefit of Fyn blockade persists after washout in mouse Alzheimer’s model. Neuropharmacology.

[28] Folch J., Petrov D., Ettcheto M., Abad S., Sanchez-Lopez E., Garcia M.L., Olloquequi J., Beas-Zarate C., Auladell C., Camins A. (2016). Current Research Therapeutic Strategies for Alzheimer’s Disease Treatment. Neural Plast..

[29] Angelopoulou E., Paudel Y.N., Julian T., Shaikh M.F., Piperi C. (2021). Pivotal Role of Fyn Kinase in Parkinson’s Disease and Levodopa-Induced Dyskinesia: A Novel Therapeutic Target?. Mol. Neurobiol..

[30] Low C.Y.B., Lee J.H., Lim F.T.W., Lee C.L., Ballard C., Francis P.T., Lai M.K.P., Tan M.G.K. (2021). Isoform-specific upregulation of FynT kinase expression is associated with tauopathy and glial activation in Alzheimer’s disease and Lewy body dementias. Brain Pathol..

[31] Chen C.D., Zeldich E., Khodr C., Camara K., Tung T.Y., Lauder E.C., Mullen P., Polanco T.J., Liu Y.Y., Zeldich D. (2019). Small Molecule Amyloid-β Protein Precursor Processing Modulators Lower Amyloid-β Peptide Levels cKit Signaling. J. Alzheimers Dis..

[32] Dubois B., Lopez-Arrieta J., Lipschitz S., Doskas T., Spiru L., Moroz S., Venger O., Vermersch P., Moussy A., Mansfield C.D. (2023). Masitinib for mild-to-moderate Alzheimer’s disease: Results from a randomized, placebo-controlled, phase 3, clinical trial. Alzheimers Res. Ther..

[33] Fagiani F., Lanni C., Racchi M., Govoni S. (2020). Targeting dementias through cancer kinases inhibition. Alzh Dement.-Trci..

[34] Vermersch P., Brieva-Ruiz L., Fox R.J., Paul F., Ramio-Torrenta L., Schwab M., Moussy A., Mansfield C., Hermine O., Maciejowski M. (2022). Efficacy and Safety of Masitinib in Progressive Forms of Multiple Sclerosis A Randomized, Phase 3, Clinical Trial. Neurol-Neuroimmunol..

[35] Sapko K., Jamroz-Wisniewska A., Rejdak K. (2022). Novel Drugs in a Pipeline for Progressive Multiple Sclerosis. J. Clin. Med..

[36] Maragakis N.J., de Carvalho M., Weiss M.D. (2023). Therapeutic targeting of ALS pathways: Refocusing an incomplete picture. Ann. Clin. Transl. Neurol..

[37] Metcalfe D.D., Baram D., Mekori Y.A. (1997). Mast cells. Physiol. Rev..

[38] Jensen B.M., Akin C., Gilfillan A.M. (2008). Pharmacological targeting of the KIT growth factor receptor: A therapeutic consideration for mast cell disorders. Brit J. Pharmacol..

[39] Kempuraj D., Mentor S., Thangavel R., Ahmed M.E., Selvakumar G.P., Raikwar S.P., Dubova I., Zaheer S., Iyer S.S., Zaheer A. (2019). Mast Cells in Stress, Pain, Blood-Brain Barrier, Neuroinflammation and Alzheimer’s Disease. Front. Cell Neurosci..

[40] Kempuraj D., Selvakumar G.P., Thangavel R., Ahmed M.E., Zaheer S., Raikwar S.P., Iyer S.S., Bhagavan S.M., Beladakere-Ramaswamy S., Zaheer A. (2017). Mast Cell Activation in Brain Injury, Stress, and Post-traumatic Stress Disorder and Alzheimer’s Disease Pathogenesis. Front. Neurosci..

[41] Traina G. (2017). Mast cells in the brain—Old cells, new target. J. Integr. Neurosci..

[42] Kempuraj D., Thangavel R., Natteru P.A., Selvakumar G.P., Saeed D., Zahoor H., Zaheer S., Iyer S.S., Zaheer A. (2016). Neuroinflammation Induces Neurodegeneration. J. Neurol. Neurosurg. Spine.

[43] Sandhu J.K., Kulka M. (2021). Decoding Mast Cell-Microglia Communication in Neurodegenerative Diseases. Int. J. Mol. Sci..

[44] Ocak U., Ocak P.E., Wang A.N., Zhang J.H., Boling W., Wu P., Mo J., Zhang T.Y., Huang L. (2019). Targeting mast cell as a neuroprotective strategy. Brain Inj..

[45] Zhang X., Shao Z.H., Xu S.T., Liu Q.L., Liu C.M., Luo Y.P., Jin L.J., Li S.G. (2021). Immune Profiling of Parkinson’s Disease Revealed Its Association With a Subset of Infiltrating Cells and Signature Genes. Front. Aging Neurosci..

[46] Kempuraj D., Selvakumar G.P., Zaheer S., Thangavel R., Ahmed M.E., Raikwar S., Govindarajan R., Iyer S., Zaheer A. (2018). Cross-Talk between Glia, Neurons and Mast Cells in Neuroinflammation Associated with Parkinson’s Disease. J. Neuroimmune Pharm..

[47] Selvakumar G.P., Ahmed M.E., Thangavel R., Kempuraj D., Dubova I., Raikwar S.P., Zaheer S., Iyer S.S., Zaheer A. (2020). A role for glia maturation factor dependent activation of mast cells and microglia in MPTP induced dopamine loss and behavioural deficits in mice. Brain Behav. Immun..

[48] Trias E., King P.H., Si Y., Kwon Y., Varela V., Ibarburu S., Kovacs M., Moura I.C., Beckman J.S., Hermine O. (2018). Mast cells and neutrophils mediate peripheral motor pathway degeneration in ALS. JCI Insight.

[49] Spiller K.J., Restrepo C.R., Khan T., Dominique M.A., Fang T.C., Canter R.G., Roberts C.J., Miller K.R., Ransohoff R.M., Trojanowski J.Q. (2018). Microglia-mediated recovery from ALS-relevant motor neuron degeneration in a mouse model of TDP-43 proteinopathy. Nat. Neurosci..

[50] González-Rodríguez P., Cheray M., Keane L., Engskog-Vlachos P., Joseph B. (2022). ULK3-dependent activation of GLI1 promotes DNMT3A expression upon autophagy induction. Autophagy.

[51] Lee Y., Jung J., Cho K.J., Lee S.K., Park J.W., Oh I.H., Kim G.J. (2013). Increased SCF/c-kit by hypoxia promotes autophagy of human placental chorionic plate-derived mesenchymal stem cells via regulating the phosphorylation of mTOR. J. Cell Biochem..

[52] O’Connell C.E., Vassilev A. (2021). Combined Inhibition of p38MAPK and PIKfyve Synergistically Disrupts Autophagy to Selectively Target Cancer Cells. Cancer Res..

[53] Zhang Z.Q., Liu X., Shen Z.L., Quan J., Lin C.W., Li X.R., Hu G. (2021). Endostatin in fibrosis and as a potential candidate of anti-fibrotic therapy. Drug Deliv..

[54] Rong Y.G., Liu M., Ma L., Du W.Q., Zhang H.S., Tian Y., Cao Z., Li Y., Ren H., Zhang C.M. (2012). Clathrin and phosphatidylinositol-4,5-bisphosphate regulate autophagic lysosome reformation. Nat. Cell Biol..

[55] Hessvik N.P., Overbye A., Brech A., Torgersen M.L., Jakobsen I.S., Sandvig K., Llorente A. (2016). PIKfyve inhibition increases exosome release and induces secretory autophagy. Cell Mol. Life Sci..

[56] Giannakopoulos P., Herrmann F.R., Bussiere T., Bouras C., Kovari E., Perl D.P., Morrison J.H., Gold G., Hof P.R. (2003). Tangle and neuron numbers, but not amyloid load, predict cognitive status in Alzheimer’s disease. Neurology.

[57] Ethell D.W. (2010). An amyloid-notch hypothesis for Alzheimer’s disease. Neuroscientist.

[58] Fang E.F., Hou Y., Palikaras K., Adriaanse B.A., Kerr J.S., Yang B., Lautrup S., Hasan-Olive M.M., Caponio D., Dan X. (2019). Mitophagy inhibits amyloid-beta and tau pathology and reverses cognitive deficits in models of Alzheimer’s disease. Nat. Neurosci..

[59] Sun H., Zhong Y., Zhu X., Liao H., Lee J., Chen Y., Ma L., Ren J., Zhao M., Tu M. (2021). A Tauopathy-Homing and Autophagy-Activating Nanoassembly for Specific Clearance of Pathogenic Tau in Alzheimer’s Disease. ACS Nano.

[60] Heckmann B.L., Teubner B.J.W., Tummers B., Boada-Romero E., Harris L., Yang M., Guy C.S., Zakharenko S.S., Green D.R. (2020). LC3-Associated Endocytosis Facilitates β-Amyloid Clearance and Mitigates Neurodegeneration in Murine Alzheimer’s Disease. Cell.

